# Reclassification of *DMD* Duplications as Benign: Recommendations for Cautious Interpretation of Variants Identified in Prenatal Screening

**DOI:** 10.3390/genes13111972

**Published:** 2022-10-28

**Authors:** Wenbin He, Guiquan Meng, Xiao Hu, Jing Dai, Jiyang Liu, Xiurong Li, Hao Hu, Yueqiu Tan, Qianjun Zhang, Guangxiu Lu, Ge Lin, Juan Du

**Affiliations:** 1 Institute of Reproductive and Stem Cell Engineering, NHC Key Laboratory of Human Stem Cell and Reproductive Engineering, School of Basic Medical Science, Central South University, Changsha 410008, China; 2 National Engineering and Research Center of Human Stem Cells, Changsha 410006, China; 3 Clinical Research Center for Reproduction and Genetics in Hunan Province, Reproductive and Genetic Hospital of CITIC-Xiangya, Changsha 410008, China; 4 Changsha Health Committee, Changsha 410006, China

**Keywords:** dystrophinopathies, *DMD* duplication, breakpoint analysis, long-read sequencing

## Abstract

Duplications are the main type of dystrophin gene (*DMD*) variants, which typically cause dystrophinopathies such as Duchenne muscular dystrophy and Becker muscular dystrophy. Maternally inherited exon duplication in *DMD* in fetuses is a relatively common finding of genetic screening in clinical practice. However, there is no standard strategy for interpretation of the pathogenicity of *DMD* duplications during prenatal screening, especially for male fetuses, in which maternally inherited pathogenic *DMD* variants more frequently cause dystrophinopathies. Here, we report three non-contiguous *DMD* duplications identified in a woman and her male fetus during prenatal screening. Multiplex ligation probe amplification and long-read sequencing were performed on the woman and her family members to verify the presence of *DMD* duplications. Structural rearrangements in the *DMD* gene were mapped by long-read sequencing, and the breakpoint junction sequences were validated using Sanger sequencing. The woman and her father carried three non-contiguous *DMD* duplications. Long-read and Sanger sequencing revealed that the woman’s father carried an intact *DMD* copy and a complex structural rearrangement of the *DMD* gene. Therefore, we reclassified these three non-contiguous *DMD* duplications, one of which is listed as pathogenic, as benign. We postulate that breakpoint analysis should be performed on identified *DMD* duplication variants, and the pathogenicity of the duplications found during prenatal screening should be interpreted cautiously for clinical prediction and genetic/reproductive counseling.

## 1. Introduction

Dystrophinopathies are a group of severe and incurable muscle disorders, including Duchenne muscular dystrophy (DMD) and Becker muscular dystrophy (BMD), with an estimated incidence ranging from 1 in 3802 to 1 in 62911 in newborn males [1,2,3]. DMD and BMD are characterized by progressive proximal muscle weakness and degeneration, resulting in wheelchair dependency and death due to respiratory failure [1]. Dystrophinopathies are X-linked recessive inherited disorders resulting from various pathogenic mutations in the dystrophin gene (*DMD*) [4]. However, there is currently no effective therapy available that can permanently restore dystrophin expression and improve the clinical phenotype [5].

The *DMD* gene is the largest known gene in humans, spanning approximately 2.4 Mb on chromosome Xp21.1 comprising of 79 exons [6]. As of 31 May 2022, more than 7100 *DMD* mutations, including exon deletions, exon duplications, and point mutations, have been described in the Leiden Open Variation Database (LOVD) and the UMD-DMD France Database. Exon duplication is the most common mutation, accounting for approximately 7% of all *DMD* pathogenic mutations [7,8]. Approximately 1 in 5224 fetuses carry maternally inherited exon duplications of the *DMD* gene [9]. However, not all maternal *DMD* duplications, including those reported as pathogenic variants in the above database, are pathogenic. Nevertheless, there is currently no standard strategy for the interpretation of the pathogenicity of *DMD* duplications in prenatal screening, especially for male fetuses.

Herein, we report a case of three non-contiguous maternally inherited *DMD* duplications identified in a male fetus during prenatal screening, which were subject to a series of molecular genetic analyses to classify the pathogenicity. This study thus provides a basis for genetic counseling and family planning for the woman and her family members, while offering an example for the appropriate strategic interpretation of *DMD* duplications in prenatal screening.

## 2. Materials and Methods

### 2.1. Patients

A 30-year-old woman (II-1) from a Chinese non-consanguineous family was the proband of our study. The patient had been diagnosed with hereditary neuropathy with liability to pressure palsies (HNPP) caused by a microdeletion in chromosome 17p12, which includes the peripheral myelin protein 22 gene (*PMP22*) and limits her ability to lift both of her upper limbs.

The proband became pregnant at the age of 29 years, and non-invasive prenatal screening (NIPS) performed at a local hospital identified a 1.42-Mb deletion of chromosome 17p12 in her male fetus (Ⅲ-1). Chromosome microarray analysis confirmed the microdeletion of 17p12 and identified two duplications of Xp21 (containing the *DMD* gene) using amniocytes. Additionally, multiplex ligation dependent probe amplification (MLPA) confirmed that two non-contiguous *DMD* duplications (exons 51–53 and exons 64–79) of the male fetus were inherited from the proband (II-1). The identified *DMD* duplication of exons 64–79 is documented as a pathogenic variant in the LOVD database [10]. No member of this family has received a diagnosis of DMD. After genetic counseling at the local hospital, the couple chose termination of the pregnancy considering the probability of dystrophinopathies rather than HNPP in the male fetus. Therefore, the couple came to our hospital for assisted reproduction and genetic counseling.

Peripheral blood was collected from the proband (II-1), her husband (II-2), and her parents (I-1 and I-2) using EDTA-K_2_ anticoagulation tubes. Written informed consent was obtained from all participants. This study was approved by the Institutional Ethics Committee of the Reproductive and Genetic Hospital of CITIC Xiangya.

### 2.2. MLPA and Quantitative Polymerase Chain Reaction (qPCR)

Genomic DNA (gDNA) was extracted from the peripheral blood samples using a QIAamp DNA Blood Midi Kit (Qiagen, Hilden, Germany), according to the manufacturer’s instructions. MLPA was performed to analyze copies of the *DMD* gene in the proband (II-1) and her parents (I-1 and I-2) using a SALSA P034/P035 DMD Kit (MRC Holland, Amsterdam, The Netherlands). Creatine kinase and creatine kinase isoenzyme levels in the peripheral blood were determined in cases where *DMD* variations were detected in the males of the family.

In addition, q-PCR was used to quantify the copy number of the *PMP22* gene in the proband (II-1) and her parents (I-1 and I-2) using an ABI 7500 instrument (Applied Biosystems, Carlsbad, CA, USA). Primers for the *PMP22* gene were designed using Primer3 (http://bioinfo.ut.ee/primer3-0.4.0/primer3/, accessed on 5 May 2022). The housekeeping gene *ALB* on chromosome 4 was used as a control. The primers used are listed in Supplementary Table S1.

### 2.3. DMD Sequencing and Analysis by Bionano Saphyr^TM^

Bionano Saphyr^TM^ optical mapping technology, performed on GrandOmics Biosciences (Beijing, China), was used to detect structural variations in the *DMD* gene in the proband (II-1) and her father (I-1). Peripheral blood samples were lysed using an RBC lysis solution, followed by treatment with protease K and lysis buffer to release DNA. The DNA was bound to nanobind disks and washed with wash buffer to remove impurities and excess reagent. After isolation from the disks, DNA was quantified using a Qubit dsDNA assay kit (Thermo Fisher Scientific, Waltham, MA, USA) [11,12].

Eligible DNA was labeled with DL-Green fluorophores using Direct Labeling Enzyme 1 (DLE-1). The labeled DNA was then stained with DNA stain to dye the backbone blue and quantified using the Qubit dsDNA HS assay kit (Thermo Fisher Scientific) [11,12,13]. The labeled and stained DNA was loaded onto a Saphyr Chip (Bionano Genomics, San Diego, CA, USA), and then loaded into the Saphyr instrument (Bionano Genomics) for optical genome mapping. Genome data were analyzed using the Bionano Solve v3.6.1 software (Bionano Genomics). Based on the de novo assembly of single molecules, *DMD*-related structural variations in the samples were determined by identifying the differences between the sample genome assembly and the reference genome assembly (hg38) [11,12,13].

### 2.4. Breakpoint Analysis and Sanger Sequencing

We analyzed the chromosome fragments and flanking sequences of the breakpoints from the structural variation analysis of the *DMD* gene. The breakpoints were confirmed using PCR and Sanger sequencing. The primers used for the PCR were designed using Primer3 and are listed in Supplementary Table S1. The mechanism underlying this chromosomal rearrangement was determined by analyzing the sequences near the breakpoints.

## 3. Results

### 3.1. MLPA and q-PCR Analysis

**Figure 1:**
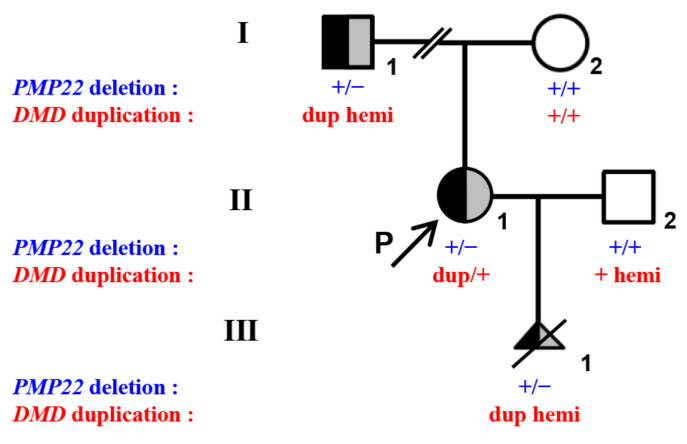
Pedigree and genotypes of a Chinese family with *DMD* duplications and *PMP22* deletion. I-1, II-1 (proband), and III-1 of the family showed heterozygous deletion in the *PMP22* gene and three discontinuous duplications in the *DMD* gene (exons 51–53, exons 64–79, and intron 55). The black-filled symbol represents individuals with *PMP22* deletion. The gray-filled symbol represents individuals with *DMD* duplications. The half-black and half-gray symbols represent heterozygous *PMP22* and *DMD* mutation carriers. The open symbols represent individuals free of *PMP22* and *DMD* variation. The arrow represents the proband (P). The slash represents the termination of pregnancy. Dup is duplication.

We first conducted a pedigree analysis in the family and evaluated the presence of duplications in the *DMD* gene in the woman (II-1) and her parents (I-1 and I-2) by MLPA. We identified two non-contiguous *DMD* duplications (four copies of exons 51–53 and 64–79) in the woman that were inherited from her father (I-1) (three copies of both exons 51–53 and 64–79). The copy number of the *DMD* gene was normal in the proband’s mother (I-2) (Figure 1 and Supplementary Figure S1). Her father showed no symptoms related to DMD/BMD such as myasthenia, amyotrophia, and pseudohypertrophy of the gastrocnemius muscle, and he had normal serum creatine kinase (118 IU/L, reference range: 38–182 IU/L) and creatine kinase isozyme (16 IU/L, reference range: 0–24 IU/L) concentrations.

Additionally, q-PCR revealed that the proband (II-1) and her father (I-1) carried two *PMP22* deletions encompassing exons 1–5 and exons 4–5, respectively. The copy number of the *PMP22* gene in the proband’s mother (I-2) was normal (Figure 1).

### 3.2. Long-Read Sequencing and Breakpoint Analysis

Given the normal levels of creatine kinase and creatine kinase isoenzyme in the proband’s father (I-1), we suspected that the discontinuous *DMD* duplications (exons 51–53 and 64–79) in the family to be benign variants. To further verify the pathogenicity of these duplications, optical genome mapping technology was used to determine the complex structural variations of the *DMD* gene in the proband’s father (I-1).

**Figure 2:**
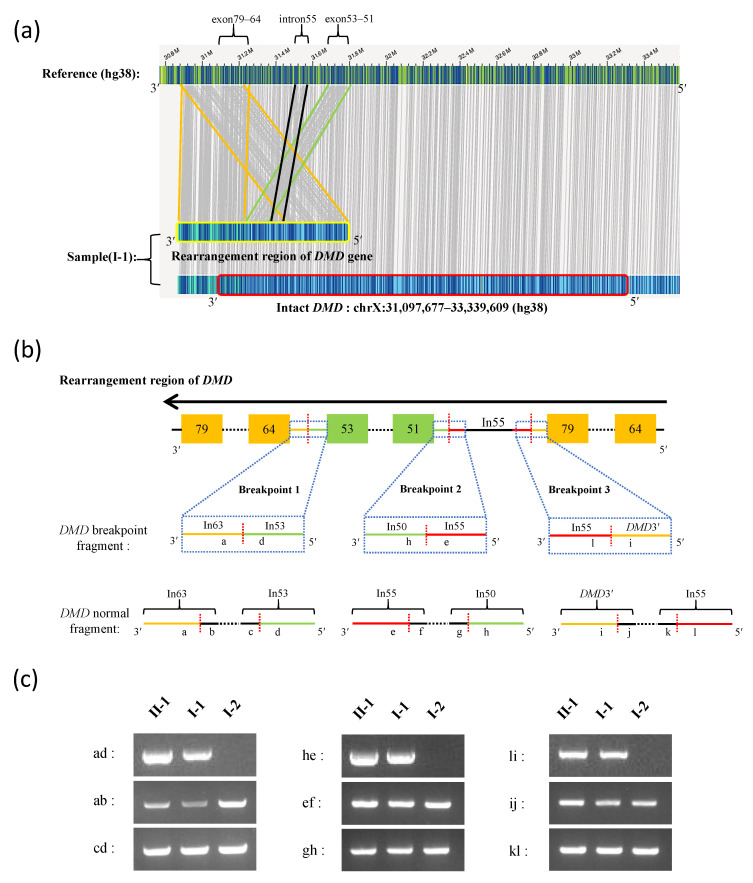
Long-read sequencing and breakpoint analysis of the *DMD* gene. (**a**) The *DMD* gene structure detected by Bionano SaphyrTM optical genome mapping. Compared with the human reference genome (hg38), the intact *DMD* gene is marked by a red border, and the rearrangement region of the *DMD* gene is marked by a yellow border. Gray vertical lines represent matching sequences between the sample *DMD* gene and the reference genome. The region between orange, black, and green lines represent *DMD* duplications in exons 79–64, intron 55, and exons 53–51, respectively. (**b**) The pattern of rearrangement in the *DMD* gene and the breakpoint structures. The orange- and green-filled boxes represent the exons. The black horizontal line represents introns. The black horizontal dashed line represents contiguous exons and introns of the *DMD* gene. The red vertical dashed line represents the breakpoint site. The blue dashed frame represents the magnified breakpoint structure, and “in” represents intron. (**c**) PCR verification of the breakpoint junction sequence and normal sequence of the *DMD* gene using gDNA from the proband (II-1) and her parents (I-1 and I-2): “ab” and “cd”, “ef” and “gh”, and “ij” and “kl” represent flanking sequence of the *DMD* gene breakpoint 1, 2, and 3, respectively; “ad”, “he”, and “li” represent *DMD* junction sequences of breakpoint 1, 2, and 3, respectively.

Long-read sequencing identified three non-contiguous *DMD* duplications (exons 51–53, exons 64–79, and intron 55) in both the proband (II-1) and her father (I-1). The father (I-1) carried three copies of *DMD* exons 51–53, three copies of *DMD* exons 64–79, and two copies of *DMD* intron 55, and the proband carried these same duplications along with another copy of the maternally inherited *DMD* gene. Optical genome mapping in comparison with the human reference genome (hg38) revealed that the proband’s father (I-1) carried an intact *DMD* gene copy (including one copy each of exons 51–53 and 64–79, and one copy of intron 55) and a complex structural rearrangement of the other *DMD* copy (Figure 2a). The complex rearrangement contained two copies of exon 64–79, one copy of intron 55, and one copy of exon 51–53, which formed three breakpoint junctions (Figure 2a,b). Unfortunately, the location of the other copy of exon 51–53 was unclear.

Breakpoint analysis showed that breakpoint 1 occurred between intron 53 and intron 63, breakpoint 2 occurred between intron 50 and intron 55, and breakpoint 3 occurred between intron 55 and the downstream region (3’ end) of the *DMD* gene (adjacent to the *TAB3* gene) (Figure 2b). PCR was used for the verification of the breakpoints for the complex structural rearrangements of the *DMD* gene in the proband (II-1) and her father (I-1). A normal structure of the *DMD* gene associated with the breakpoint regions was detected in both the proband (II-1) and her father (I-1), which is consistent with the genome mapping results (Figure 2c).

The sequences of the breakpoint junctions were analyzed to determine the mechanism of duplication. Sanger sequencing revealed that the junction sequences of breakpoints 1, 2, and 3 were TAATGAAAAG|GTTTTGTTTT, CAACAACAGA|CATTTAGATG, and AATATTTTTA|TCTTTGAAGC, respectively (Table 1, Supplementary Figure S2). Microhomologies were found near breakpoint 1 and 2, indicating that the rearrangement was caused by microhomology-mediated replication-dependent recombination (MMRDR). Rearrangement in breakpoint 3 was due to a non-homologous end-joining (NHEJ) repair. The breakpoint sites and regions of these three breakpoints are listed in Table 1. The presence of an intact *DMD* gene in the proband’s father (I-1) was confirmed by long-read sequencing; however, the precise structure of the gene with complex structural rearrangement remains unclear as the location of the duplication encompassing exons 51–53 could not be confirmed.

## 4. Discussion

In this study, MLPA, Sanger sequencing, and long-read sequencing were employed to interpret and classify the pathogenicity of three non-contiguous *DMD* duplications (exons 51–53, exons 64–79, and intron 55). Although the *DMD* duplication at exons 64–79 is reported as a pathogenic variant in the LOVD database [14], we reclassified the three non-contiguous *DMD* duplications identified in this study as benign variants because the *DMD* gene remains intact. To the best of our knowledge, this is the first report to classify the three non-contiguous *DMD* duplications identified in this study as benign.

Exon duplication is a common type of *DMD* pathogenic mutation that occurs at a frequency of up to 8.28% in Chinese patients with DMD/BMD [7]. In the majority of these cases, the extra copy is located within the *DMD* gene, resulting in disruption of its function; thus, exon duplication in the *DMD* gene is usually pathogenic [15]. However, the location of the duplication breakpoints differs among affected families; therefore, the same duplication may be pathogenic in one family but benign in another [16,17]. In a previous study, fluorescent multiplex qPCR and MLPA identified three non-contiguous duplications of exons 44–48, 51–59, and 64–79 in a patient with DMD, which were classified as pathogenic [14]. However, breakpoint analysis was not performed to confirm whether the *DMD* duplications were within the *DMD* gene. In the present study, the proband and her father carried three non-contiguous *DMD* duplications in exons 51–53, exons 64–79, and intron 55. As the father did not exhibit any symptoms related to DMD/BMD, we used long-read sequencing and breakpoint analysis, showing a complex structural rearrangement of the *DMD* gene and an intact *DMD* gene, and all extra copies were outside the *DMD* gene region. Thus, the non-contiguous *DMD* duplications may be benign in this family. Therefore, we suggest that any extra copy duplications identified in screening should be mapped along with breakpoint analysis for individuals without a family history of DMD or BMD to assess variant pathogenicity.

Several studies have analyzed the molecular characterization of *DMD* complex rearrangements in patients with DMD. Baskin et al. [18] used a combination of MLPA, mRNA transcript analysis, array comparative hybridization arrays, and breakpoint sequence analysis to analyze the structure rearrangements and breakpoints of the *DMD* gene. Luce et al. [19] used a multi-technique algorithm, including MLPA, microarrays, and next-generation whole-genome sequencing for the molecular characterization of complex structural variants in the *DMD* gene. Recently, long-read sequencing and Sanger sequencing revealed that the duplication of exons 56–61 in the *DMD* gene was a tandem repeat in a patient with DMD and that duplication occurred outside the *DMD* gene in an asymptomatic male [16]. We used long-read sequencing and breakpoint analysis to illustrate the molecular characteristics of three non-contiguous *DMD* duplications, which proved to be benign. Therefore, we suggest that interpretation of the pathogenicity of complex structural variations should be based on assessments using different technologies in different laboratories.

Segregation analysis is widely used to determine whether a given variant underlies the distribution of a disease in a family. According to the American College of Medical Genetics and Genomics standards and guidelines for the interpretation of variants, segregation data provide important evidence for variant classification [20]. In our study, non-contiguous *DMD* duplications were identified in the woman and her male fetus with a negative family history of DMD. Unfortunately, the proband initially refused segregation analysis and decided to terminate the pregnancy at a local hospital. However, pedigree analysis and MLPA performed at our hospital for subsequent genetic and reproductive counseling revealed that non-contiguous *DMD* duplications found in the male fetus were also present in an asymptomatic male (I-1) in the family (the proband’s father), indicating that the variants in question may be benign. Thus, segregation analysis should ideally be conducted to assess the pathogenicity of variants identified in genetic screening, especially in prenatal testing and carrier screening.

At least five mechanisms have been reported to be involved in genomic recombination: (1) homologous recombination, (2) NHEJ, (3) MMRDR, (4) telomere healing, and (5) long interspersed element-1-mediated retrotransposition [21,22]. NHEJ and MMRDR are the main mechanisms of *DMD* recombination [22,23]. In our study, three definite breakpoints and rearrangements were identified by long-read sequencing. Analysis of the junction sequence of the breakpoints revealed that microhomologies were in two rearrangements (breakpoint 1 and breakpoint 2), indicating that MMRDR is responsible for the rearrangements. The other rearrangement was due to NHEJ. In addition, the three breakpoints occurred in intron regions, in accordance with previously reported results [22]. Therefore, we postulate that both NHEJ and MMRDR are important mechanisms underlying *DMD* rearrangements and that breakpoints are usually located in intron regions.

NIPS is an effective method for detecting aneuploidies, and expanded NIPS panels and fetal fraction amplification within NIPS can detect 1-Mb deletions and 2-Mb duplications [24,25]. Chromosomal copy number variations (CNVs) are frequently detected in fetuses during prenatal screening and can be inherited from a parent who is unaffected, has mild clinical symptoms, or may also occur de novo [26]. In our study, 1.42-Mb deletion of 17p12 was identified in a male fetus using NIPS, and two additional duplications of Xp21 were found by invasive prenatal diagnosis. The deletion encompasses the *PMP22* gene, which is responsible for HNPP in the proband [27], and the duplications encompass the *DMD* gene. Furthermore, CNVs in these two genes are the main pathogenic mutations, among which, the deletion in the *PMP22* gene and one *DMD* duplication have been reported as pathogenic variants. HNPP resulting from *PMP22* gene deletion is a clinical disorder with mild symptoms and generally does not affect the carrier’s quality of life [28], as in the case of the proband in our study; however, the proband decided to abort the male fetus considering the potential risk of dystrophinopathies. Subsequently, segregation analysis and long-read sequencing showed that the *DMD* duplications identified in this family were benign variants, suggesting that the aborted fetus was not likely to have suffered from dystrophinopathies. Therefore, CNVs, particularly duplications identified during prenatal screening, should be treated with caution.

## 5. Conclusions

In conclusion, this is the first report of the reclassification of three non-contiguous *DMD* duplications identified in prenatal screening as benign variants in a Chinese family, with one of the duplications being recorded as pathogenic in the LOVD database. Therefore, we suggest that breakpoint analysis should be performed on identified *DMD* duplication variants, and the pathogenicity of *DMD* duplications identified in prenatal screening should be cautiously interpreted for clinical outcome prediction. This study also highlights the importance of clinical evaluation and precise molecular genetic analyses for clinical prediction by variant classification.

## Figures and Tables

**Table 1:** Characteristics of *DMD* breakpoint junction sequences.

ID	Breakpoint Junction Sequence (5′-3′)	Breakpoint 5′ Region	Breakpoint 5′ Sequence	Breakpoint 5′ Site	Breakpoint 3′ Region	Breakpoint 3′ Sequence	Breakpoint 3′ Site
Breakpoint 1	TAATGAAAAG|GTTTTGTTTT	Intron 53	TAATGAAAAG	ChrX: 31,674,049	Intron 63	GTTTTGTTTT	ChrX: 31,260,251
Breakpoint 2	CAACAACAGA|CATTTAGATG	Intron 55	CAACAACAGA	ChrX: 31,513,138	Intron 50	CATTTAGATG	ChrX: 31,811,202
Breakpoint 3	AATATTTTTA|TCTTTGAAGC	*DMD*3′	AATATTTTTA	ChrX: 30,880,778	Intron 55	TCTTTGAAGC	ChrX: 31,570,889

## Data Availability

The datasets generated during the current study are available upon request.

## Supplementary Materials

The following supporting information can be downloaded at: https://www.mdpi.com/article/10.3390/genes13111972/s1, Figure S1: Duplications of exons 51–53 and 64–79 in *DMD* were confirmed by MLPA analysis in proband and her parents; Figure S2: Three breakpoints of *DMD* were verified by Sanger sequencing; Table S1: Supplementary Table S1. Primers used in quantitative real-time PCR and breakpoint analysis.

## References

[1] Flanigan K.M. (2014). Duchenne and Becker Muscular Dystrophies. Neurol. Clin..

[2] Emery A.E. (1991). Population Frequencies of Inherited Neuromuscular Diseases—A World Survey. Neuromuscul. Disord..

[3] Mendell J.R., Shilling C., Leslie N.D., Flanigan K.M., al-Dahhak R., Gastier-Foster J., Kneile K., Dunn D.M., Duval B., Aoyagi A. (2012). Evidence-Based Path to Newborn Screening for Duchenne Muscular Dystrophy. Ann. Neurol..

[4] Duan D., Goemans N., Takeda S., Mercuri E., Aartsma-Rus A. (2021). Duchenne Muscular Dystrophy. Nat. Rev. Dis. Primers.

[5] Happi Mbakam C., Lamothe G., Tremblay J.P. (2022). Therapeutic Strategies for Dystrophin Replacement in Duchenne Muscular Dystrophy. Front. Med..

[6] Den Dunnen J.T., Grootscholten P.M., Dauwerse J.G., Walker A.P., Monaco A.P., Butler R., Anand R., Coffey A.J., Bentley D.R., Steensma H.Y. (1992). Reconstruction of the 2.4 Mb Human DMD-Gene by Homologous YAC Recombination. Hum. Mol. Genet..

[7] Kong X., Zhong X., Liu L., Cui S., Yang Y., Kong L. (2019). Genetic Analysis of 1051 Chinese Families with Duchenne/Becker Muscular Dystrophy. BMC Med. Genet..

[8] Zamani G., Hosseinpour S., Ashrafi M.R., Mohammadi M., Badv R.S., Tavasoli A.R., Akbari M.G., Bereshneh A.H., Malamiri R.A., Heidari M. (2022). Characteristics of Disease Progression and Genetic Correlation in Ambulatory Iranian Boys with Duchenne Muscular Dystrophy. BMC Neurol..

[9] Brison N., Storms J., Villela D., Claeys K.G., Dehaspe L., de Ravel T., De Waele L., Goemans N., Legius E., Peeters H. (2019). Maternal Copy-Number Variations in the DMD Gene as Secondary Findings in Noninvasive Prenatal Screening. Genet. Med..

[10] Fokkema I.F.A.C., Kroon M., López Hernández J.A., Asscheman D., Lugtenburg I., Hoogenboom J., den Dunnen J.T. (2021). The LOVD3 Platform: Efficient Genome-Wide Sharing of Genetic Variants. Eur. J. Hum. Genet..

[11] Goldrich D.Y., LaBarge B., Chartrand S., Zhang L., Sadowski H.B., Zhang Y., Pham K., Way H., Lai C.-Y.J., Pang A.W.C. (2021). Identification of Somatic Structural Variants in Solid Tumors by Optical Genome Mapping. J. Pers. Med..

[12] Stence A.A., Thomason J.G., Pruessner J.A., Sompallae R.R., Snow A.N., Ma D., Moore S.A., Bossler A.D. (2021). Validation of Optical Genome Mapping for the Molecular Diagnosis of Facioscapulohumeral Muscular Dystrophy. J. Mol. Diagn..

[13] Barseghyan H., Tang W., Wang R.T., Almalvez M., Segura E., Bramble M.S., Lipson A., Douine E.D., Lee H., Délot E.C. (2017). Next-Generation Mapping: A Novel Approach for Detection of Pathogenic Structural Variants with a Potential Utility in Clinical Diagnosis. Genome Med..

[14] Carsana A., Frisso G., Intrieri M., Tremolaterra M.R., Savarese G., Scapagnini G., Esposito G., Santoro L., Salvatore F. (2010). A 15-Year Molecular Analysis of DMD/BMD: Genetic Features in a Large Cohort. Front. Biosci..

[15] Aartsma-Rus A., den Dunnen J.T. (2019). Phenotype Predictions for Exon Deletions/Duplications: A User Guide for Professionals and Clinicians Using Becker and Duchenne Muscular Dystrophy as Examples. Hum. Mutat..

[16] Bai Y., Liu J., Xu J., Sun Y., Li J., Gao Y., Liu L., Jia C., Kong X., Wang L. (2022). Long-Read Sequencing Revealed Extragenic and Intragenic Duplications of Exons 56-61 in DMD in an Asymptomatic Male and a DMD Patient. Front. Genet..

[17] White S.J., Aartsma-Rus A., Flanigan K.M., Weiss R.B., Kneppers A.L.J., Lalic T., Janson A.a.M., Ginjaar H.B., Breuning M.H., den Dunnen J.T. (2006). Duplications in the DMD Gene. Hum. Mutat..

[18] Baskin B., Stavropoulos D.J., Rebeiro P.A., Orr J., Li M., Steele L., Marshall C.R., Lemire E.G., Boycott K.M., Gibson W. (2014). Complex Genomic Rearrangements in the Dystrophin Gene Due to Replication-Based Mechanisms. Mol. Genet. Genom. Med..

[19] Luce L., Abelleyro M.M., Carcione M., Mazzanti C., Rossetti L., Radic P., Szijan I., Menazzi S., Francipane L., Nevado J. (2021). Analysis of Complex Structural Variants in the *DMD* Gene in One Family. Neuromuscul. Disord..

[20] Richards S., Aziz N., Bale S., Bick D., Das S., Gastier-Foster J., Grody W.W., Hegde M., Lyon E., Spector E. (2015). Standards and Guidelines for the Interpretation of Sequence Variants: A Joint Consensus Recommendation of the American College of Medical Genetics and Genomics and the Association for Molecular Pathology. Genet. Med..

[21] Chen J.-M., Cooper D.N., Férec C., Kehrer-Sawatzki H., Patrinos G.P. (2010). Genomic Rearrangements in Inherited Disease and Cancer. Semin. Cancer Biol..

[22] Ling C., Dai Y., Fang L., Yao F., Liu Z., Qiu Z., Cui L., Xia F., Zhao C., Zhang S. (2020). Exonic Rearrangements in DMD in Chinese Han Individuals Affected with Duchenne and Becker Muscular Dystrophies. Hum. Mutat..

[23] Ishmukhametova A., Khau Van Kien P., Méchin D., Thorel D., Vincent M.-C., Rivier F., Coubes C., Humbertclaude V., Claustres M., Tuffery-Giraud S. (2012). Comprehensive Oligonucleotide Array-Comparative Genomic Hybridization Analysis: New Insights into the Molecular Pathology of the DMD Gene. Eur. J. Hum. Genet..

[24] Maya I., Salzer Sheelo L., Brabbing-Goldstein D., Matar R., Kahana S., Agmon-Fishman I., Klein C., Gurevitch M., Basel-Salmon L., Sagi-Dain L. (2022). Residual Risk for Clinically Significant Copy Number Variants in Low-Risk Pregnancies, Following Exclusion of Noninvasive Prenatal Screening-Detectable Findings. Am. J. Obstet. Gynecol..

[25] Advani H.V., Barrett A.N., Evans M.I., Choolani M. (2017). Challenges in Non-Invasive Prenatal Screening for Sub-Chromosomal Copy Number Variations Using Cell-Free DNA. Prenat. Diagn..

[26] Shaffer L.G., Bejjani B.A., Torchia B., Kirkpatrick S., Coppinger J., Ballif B.C. (2007). The Identification of Microdeletion Syndromes and Other Chromosome Abnormalities: Cytogenetic Methods of the Past, New Technologies for the Future. Am. J. Med. Genet. C Semin. Med. Genet..

[27] Van Paassen B.W., van der Kooi A.J., van Spaendonck-Zwarts K.Y., Verhamme C., Baas F., de Visser M. (2014). PMP22 Related Neuropathies: Charcot-Marie-Tooth Disease Type 1A and Hereditary Neuropathy with Liability to Pressure Palsies. Orphanet. J. Rare Dis..

[28] Casasnovas C., Banchs I., De Jorge L., Antónia Albertí M., Martínez-Campo Y., Povedano M., Montero J., Volpini V. (2012). A Novel Small Deletion in PMP22 Causes a Mild Hereditary Neuropathy with Liability to Pressure Palsies Phenotype. Muscle Nerve.

