# Supplementary material for: Reclassification of DMD Duplications as Benign: Recommendations for Cautious Interpretation of Variants Identified in Prenatal Screening
Source: Genes (Basel). 2022 Oct 28;13(11):1972. doi: 10.3390/genes13111972 (PMC9690433; doi:10.3390/genes13111972)
Supplement: Supplementary file 1 [file genes-13-01972-s001.zip › Supplementary Files/Supplementary Figure legends.pdf]

Supplementary Figure Legends

Supplementary Figure S1. Duplications of exons 51–53 and 64–79 in *DMD* were confirmed by MLPA analysis in proband and her parents.

MLPA revealed *DMD* duplications of exons 51–53 and 64–79 in the proband (II-1) and her father (I-1). Four copies and three copies of exons 51–53 and 64–79 were found in the proband (II-1) and her father (I-1), respectively. The copy numbers of *DMD* were normal in proband's mother (I-2). MLPA analysis of the proband and her mother used a normal female as normal control, whereas that of her father used a normal male as normal control.

Supplementary Figure S2. Three breakpoints of *DMD* were verified by Sanger sequencing.

Three breakpoints of the *DMD* identified by long-read sequencing were confirmed by Sanger sequencing. The “ab” and “cd”, “ef” and “gh”, “ij” and “kl” represent flanking sequence of *DMD* breakpoint 1, 2 and 3, respectively; “ad”, “he” and “li” represent *DMD* junction sequences of breakpoint 1, 2 and 3, respectively. The red vertical dashed line represents the breakpoint site. “in” represents intron.
