# Supplementary material for: Reclassification of DMD Duplications as Benign: Recommendations for Cautious Interpretation of Variants Identified in Prenatal Screening
Source: Genes (Basel). 2022 Oct 28;13(11):1972. doi: 10.3390/genes13111972 (PMC9690433; doi:10.3390/genes13111972)
Supplement: Supplementary file 1 [file genes-13-01972-s001.zip › Supplementary Files/Supplementary Table S1.pdf]

Supplementary Table S1. Primers used in quantitative real-time PCR and breakpoint analysis

Primer ID	Sequence (5′ to 3′)
PMP22-QE1-F:	TGGAAGAAGGGGTTACGCTGTT
PMP22-QE1-R:	GGGGGTGGGAAATTAGGCAATT
PMP22-QE2-F:	CTGGTGCTGCTGTTCGTCTC
PMP22-QE2-R:	AACACAGTCCTGAACCAGCA
PMP22-QE3-F:	AGCAATGGATCGTGGGCAA
PMP22-QE3-R:	CAAAACCAGCCTCACCGTTT
PMP22-QE4-F:	GCCACCATGATCCTGTCGAT
PMP22-QE4-R:	CATTCCGCAGACTTGGATGC
PMP22-QE5-F:	GAGTGGCATCTCAACTCGGAT
PMP22-QE5-R:	CGCGTTTCCGCAAGATCAC
DMD-63-a	CATTCACAGAAATGCAGGCT
DMD-63-b	GTGTGAAACCAAGACAACCC
DMD-53-c	AGTTACCTGGGGAAACTAGC
DMD-53-d	GGCAGCTATTTGAGTGTCTC
DMD-55-e	CCCACTGAGAACTCCACTTGA
DMD-55-f	CTGTGAAGAAAGGCATTGGTAG
DMD-50-g	TTCCATGAAGGCAGTAGGGG
DMD-50-h	AAGCCTTCCATTTTGAGCGG
DMD-3′-i	GGATAAGGAATGGGAGAACTGGT
DMD-3′-j	CAATAGCAGGGAAATGGTGTGC
DMD-55-k	TGCCCAGAGAAATTGTTGAGA
DMD-55-l	TTCCCTTTTCGGGCCAGTAT
