# Supplementary figures and images for: Reclassification of DMD Duplications as Benign: Recommendations for Cautious Interpretation of Variants Identified in Prenatal Screening
Source: Genes (Basel). 2022 Oct 28;13(11):1972. doi: 10.3390/genes13111972 (PMC9690433; doi:10.3390/genes13111972)

II-1:

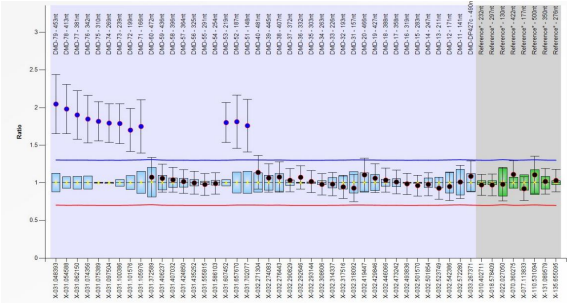

I-1:

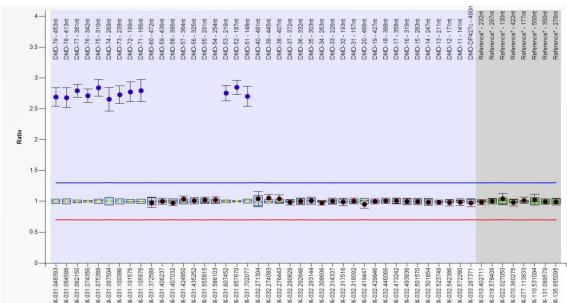

I-2:

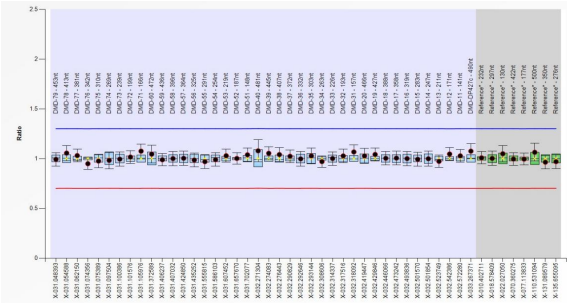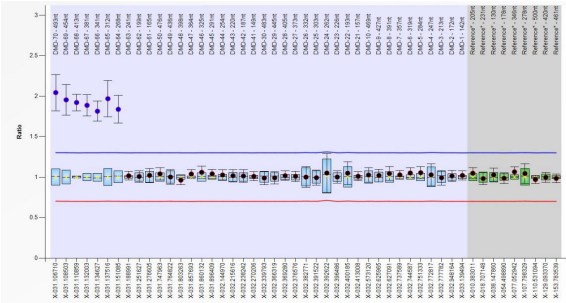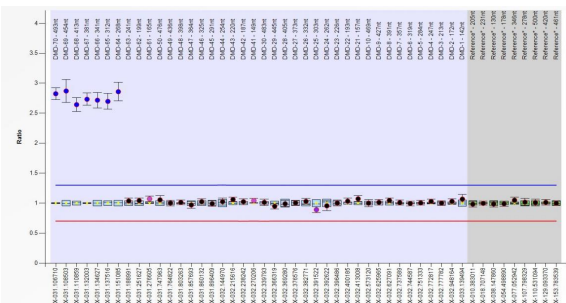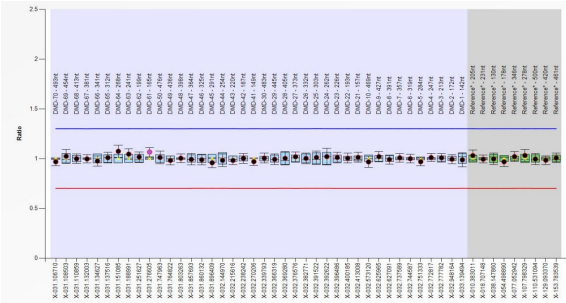

Supplement: Supplementary file 1 [file genes-13-01972-s001.zip › Supplementary Files/Supplementary Figure S1.pdf]

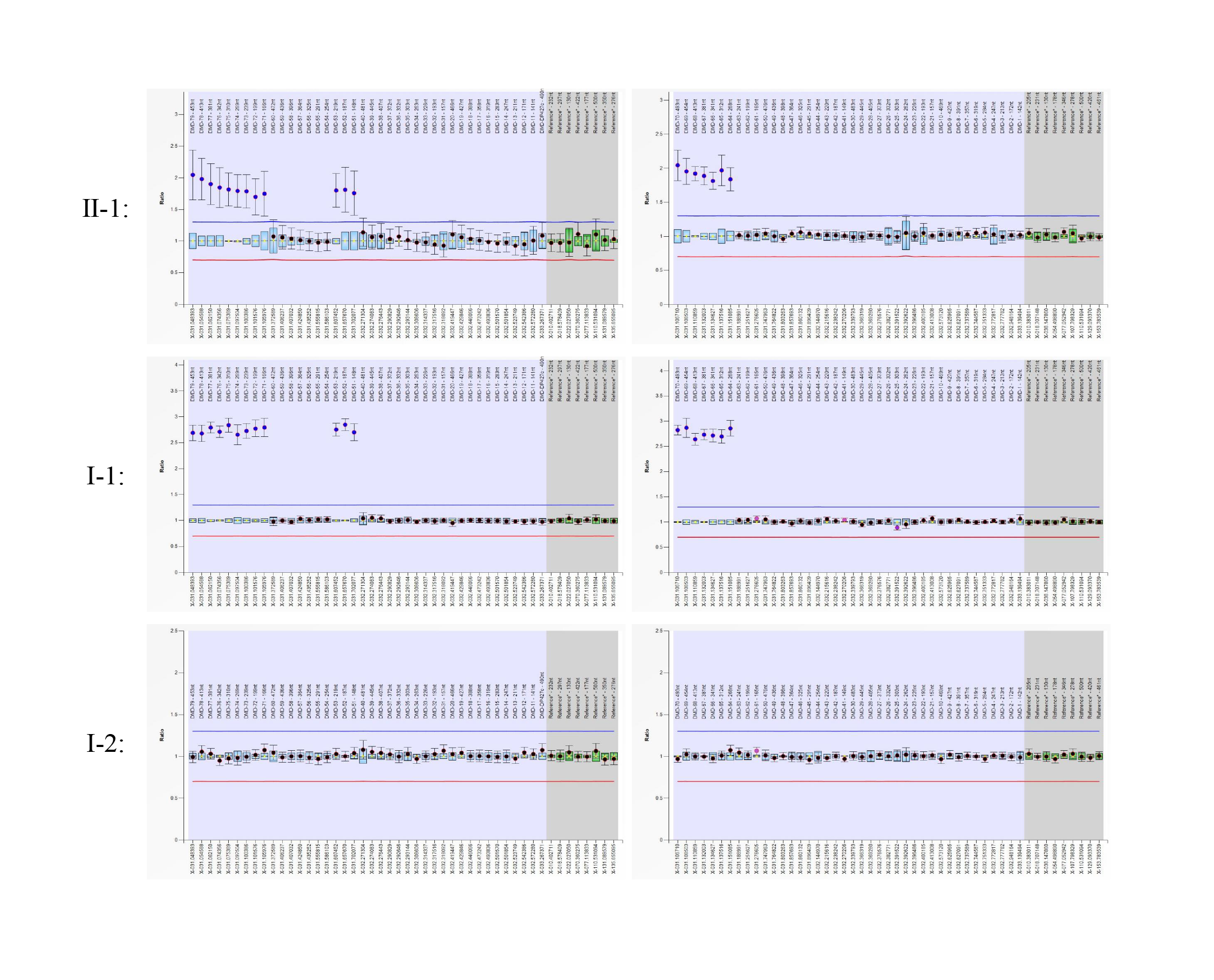

Supplement: Supplementary file 1 [file genes-13-01972-s001.zip › Supplementary Files/Supplementary Figure S1.tif]

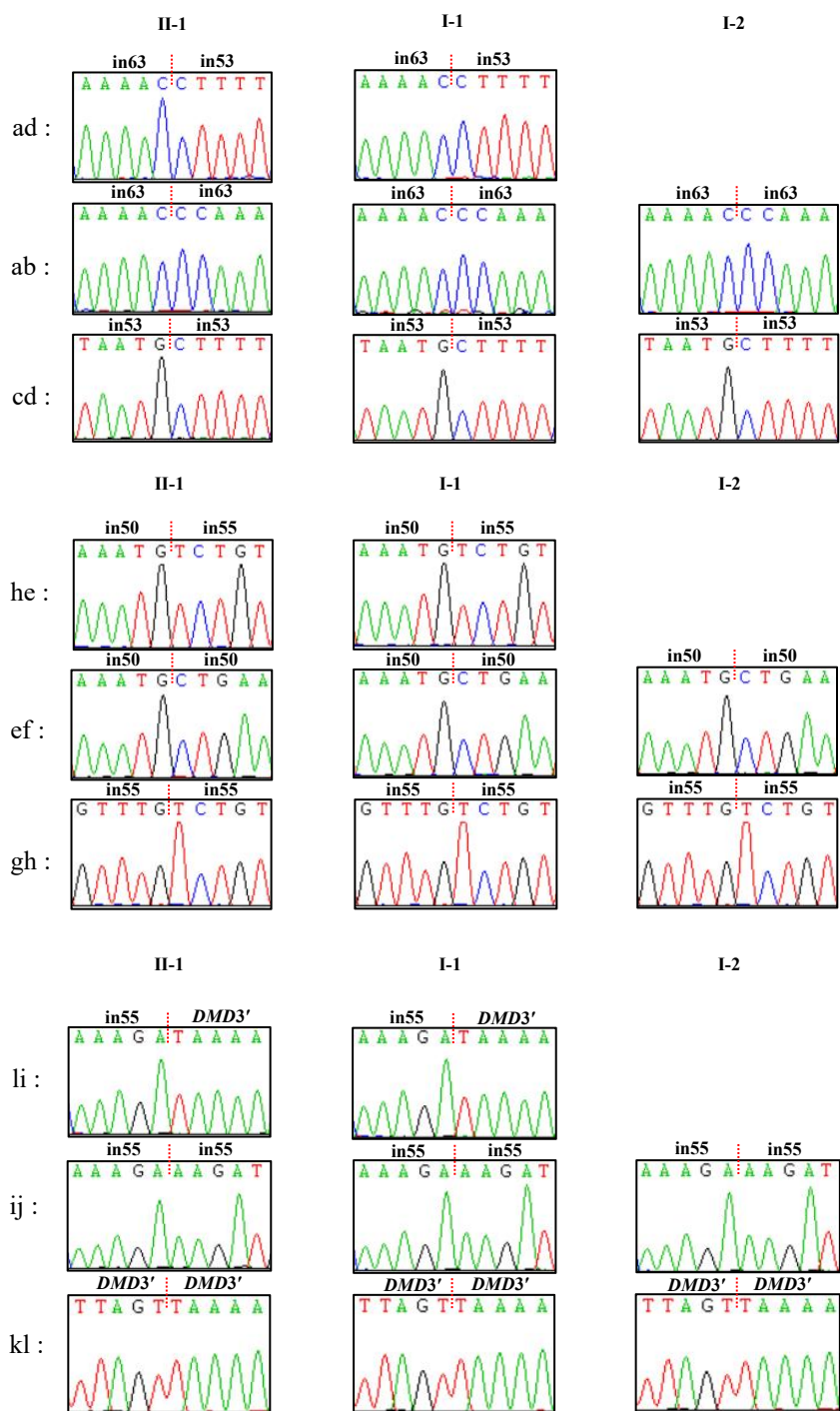

Supplement: Supplementary file 1 [file genes-13-01972-s001.zip › Supplementary Files/Supplementary Figure S2.pdf]

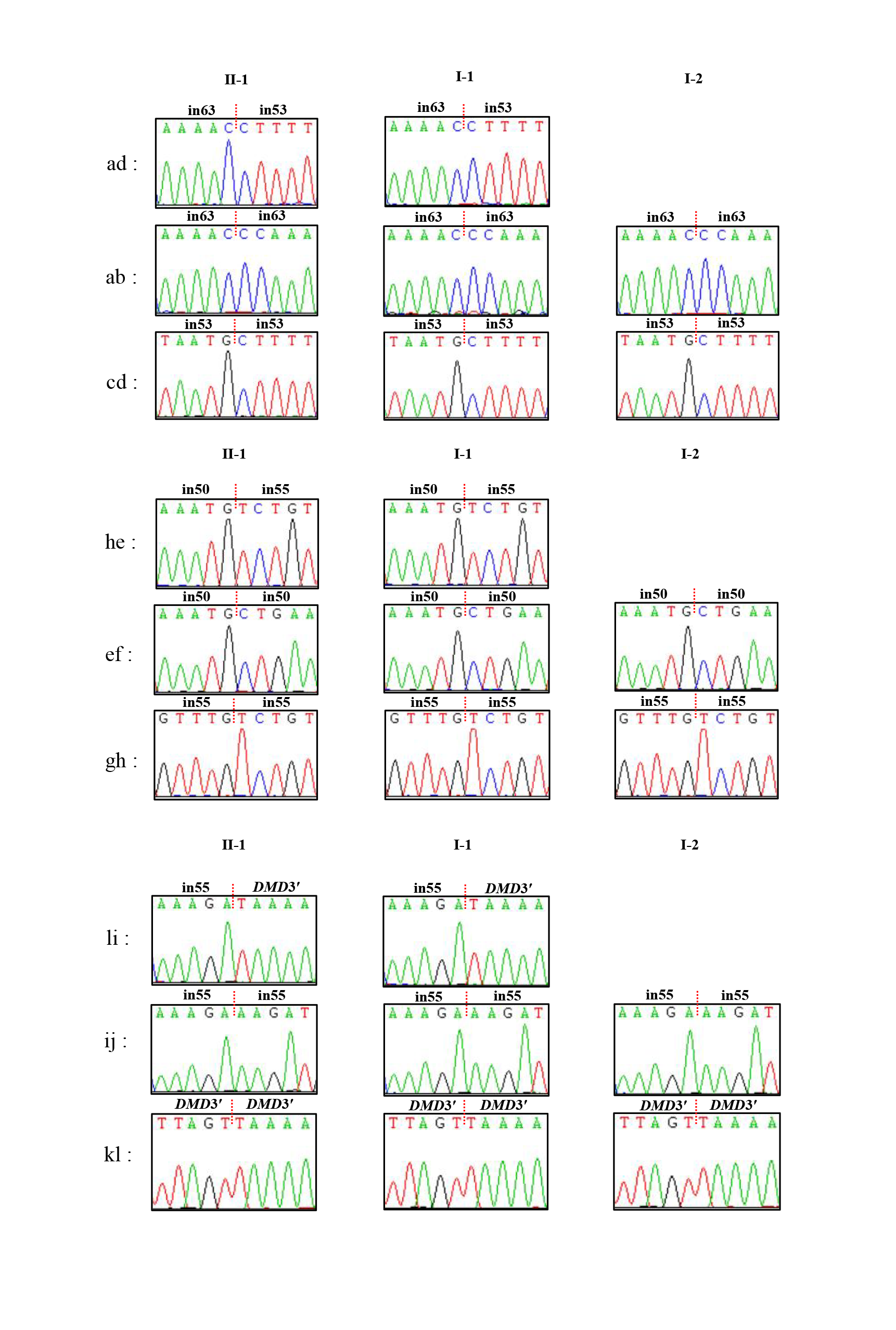

Supplement: Supplementary file 1 [file genes-13-01972-s001.zip › Supplementary Files/Supplementary Figure S2.tif]
